# Community priorities for obesity prevention among low-income adults in Kuala Lumpur: a discrete choice experiment

**DOI:** 10.1093/heapro/daac156

**Published:** 2022-11-11

**Authors:** Erica Kocher, Dallas Wood, Shiang-Cheng Lim, Angie Jackson-Morris, Ishu Kataria, Carrie Ngongo, Zhi Sham, Arunah Chandran, Rachel Nugent, Feisul Idzwan Mustapha

**Affiliations:** Global Health Division, Center for Global Noncommunicable Diseases, RTI International, 3040 East Cornwallis Road, Research Triangle Park, NC 27709, USA; Center for Applied Economics and Strategy, 3040 East Cornwallis Road, Research Triangle Park, NC 27709, USA; RTI International, Global Health Division, Suite 5.2 & 5.3, Level 5, Nucleus Tower, Jalan PJU 7/2 Mutiara Damansara, 47820, Petaling Jaya, Selangor, Malaysia; Center for Applied Economics and Strategy, 3040 East Cornwallis Road, Research Triangle Park, NC 27709, USA; Global Health Division, RTI International, 6th Floor, Commercial Tower, Pullman Hotel, Aerocity New Delhi 100037, India; Center for Applied Economics and Strategy, 3040 East Cornwallis Road, Research Triangle Park, NC 27709, USA; RTI International, Global Health Division, Suite 5.2 & 5.3, Level 5, Nucleus Tower, Jalan PJU 7/2 Mutiara Damansara, 47820, Petaling Jaya, Selangor, Malaysia; Non-Communicable Disease (NCD) Section, Disease Control Division, Ministry of Health, Level 2, Block E2, Complex E, Federal Government Administration Centre, 62590 Putrajaya, Malaysia; Center for Applied Economics and Strategy, 3040 East Cornwallis Road, Research Triangle Park, NC 27709, USA; Non-Communicable Disease (NCD) Section, Disease Control Division, Ministry of Health, Level 2, Block E2, Complex E, Federal Government Administration Centre, 62590 Putrajaya, Malaysia

**Keywords:** non-communicable diseases, obesity, nutrition, community health promotion, disease prevention

## Abstract

Non-communicable diseases and associated risk factors, such as obesity, are prevalent and increasing in Malaysia. To address this burden and the heightened vulnerability of low-income communities to these risk factors, the Better Health Programme Malaysia conducted a partial-profile discrete choice experiment (DCE) to inform the design of a community-based obesity-prevention programme. The DCE survey was conducted with community members (*n* = 1453) from three publicly supported low-cost, high-rise flat complexes in urban Kuala Lumpur. In the survey, community members were asked to choose between different sets of potential evidence-based interventions for obesity prevention. Their responses to these choice tasks were analysed to quantify preferences for these different health interventions using a random utility maximization model. Based on these results, we determined participants’ relative prioritization of the different options. The most preferred interventions were those that reduced the price of fruit and vegetables; altered cooking practices at restaurants and food vendors to reduce salt, sugar and oil; and offered reward incentives for completing online educational activities. Community members did not prioritize several evidence-based interventions, including changes to product placement or product labelling, suggesting that these effective approaches may be less familiar or simply not preferred by respondents. The DCE enabled the clear articulation of these community priorities for evidence-based interventions that focus on the supply and promotion of affordable healthy foods within the local food environment, as well as community demand for healthier food options.

## INTRODUCTION

Non-communicable diseases (NCDs), including cardiovascular disease and diabetes, are the leading causes of death worldwide, with burden projected to rise in coming decades (Global Burden of Disease Collaborative Network, 2021). Obesity is a key risk factor for NCDs and is itself associated with factors such as a sedentary lifestyle and a calorie-rich diet (World Health Organization, 2021). Although commonly viewed as the result of individual behavioural choices, NCD risk factors have important environmental determinants, including the neighbourhoods in which people live (Lakerveld and Mackenbach, 2017). Studies have shown that grocery store or market access is negatively associated with obesity, while fast food availability is positively associated with obesity (Cobb *et al.*, 2015). Proximity to recreation facilities and built spaces for physical activity is associated with greater physical activity for all age groups (Sallis *et al.*, 2012).

In Malaysia, the burden of NCDs is high and has continued to increase in recent years, accounting for an estimated 73.8% of deaths and 73.6% of disability-adjusted life years lost in 2019 (Global Burden of Disease Collaborative Network, 2021). The 2019 Malaysia National Health and Morbidity Survey estimated 18.3% of Malaysians had raised blood glucose and that the prevalence of obesity among adults was 30.4% (Institute for Public Health, 2020).

People of low socio-economic status are often disproportionately affected by environmental obesity risks through what has been termed an ‘obesogenic environment’ (Swinburn and Egger, 2002). Their neighbourhoods may not facilitate access to healthy, affordable foods, or to safe spaces for recreation (Cheung *et al.*, 2021; Wilson *et al.*, 2004). People on low incomes are likely to work long hours, reducing the time available for food preparation and physical activity (O’Donoghue *et al.*, 2018). In comparison with wealthier populations, low-income populations face both greater exposure to obesogenic environments and have fewer economic resources to overcome challenges.

In Malaysia, the lowest 40% of households on the economic spectrum (the ‘bottom 40’ or B40) have worse NCD risk factor outcomes. In 2019, the Department of Statistics Malaysia categorized the B40 group in Kuala Lumpur as having a monthly household income of less than RM 9150 (~USD 2200) (Department of Statistics, Malaysia, 2020). Obesity prevalence is higher among the low-income Kuala Lumpur population than nationally (Andoy-Galvan *et al.*, 2020). The B40 have the highest prevalence of physical inactivity and inadequate consumption of fruits and vegetables (Institute for Public Health, 2020). A 2020 survey estimated 89.5% of B40 adults had inadequate intake of fruits and vegetables (Eng *et al.*, 2022). Consistent with the global literature, in a cross-sectional study of B40 women in urban Kuala Lumpur, frequent eating out was found to be independently associated with poorer diet quality (Karupaiah *et al.*, 2013).

Effective health promotion requires interventions to reduce obesogenic environments. As Tones and Tilford outline, health promotion is necessarily a combination of health education and supporting environmental and organizational interventions (Tones and Tilford, 2001). Efforts can include both structural changes and behavioural ‘nudges’ to help make healthy options more available, accessible and appealing (Brug and Lenthe, 2005). Nudges are design features that aim to subtly guide individuals towards healthy options, such as visible placement of healthy foods at grocery stores. A 2016 meta-analysis estimated that nudges are associated with an average 15.3% increase in the frequency of healthy choices related to diet and nutrition (Arno and Thomas, 2016). Nudges associated with foods, including changes to prices, placement and labelling, have proven effective at altering individual purchasing and consumption (Afshin *et al.*, 2017; Bucher *et al.*, 2016; Cawley *et al.*, 2015; Economos *et al.*, 2013; van der Molen *et al.*, 2021; Wilson *et al.*, 2016).

It is critical to work with target communities to select appropriate nudges and environmental interventions for the desired context, or the effects of the intervention can be negatively affected or exacerbate existing disparities (Mizdrak *et al.*, 2015; van der Molen *et al.*, 2021). Discrete choice experiments (DCEs) offer one method to elicit individual preferences for potential interventions, enabling appropriate tailoring of health promotion efforts. DCE is a method of stated preference assessment that uses a survey to ask individuals to make choices between options. Responses are analysed using statistical methods to quantify respondents’ relative preference for potential interventions (Mangham *et al.*, 2009). DCEs have been used in a range of sectors, including in the design of health services and health promotion interventions (Abdel-All *et al.*, 2019; Moor *et al.*, 2020; Agarwal *et al.*, 2021).

The Better Health Programme Malaysia (BHP Malaysia) aimed to improve health literacy and alter the obesogenic environment in B40 communities in urban Kuala Lumpur through community-based health promotion interventions co-created and co-implemented with community members, community health volunteers (CHVs) and local businesses (Kataria *et al.*, 2020). BHP Malaysia included formative research to understand the needs, preferences and starting context of the participating communities in order to tailor the implementation plan to the local context. Formative research included a community knowledge, attitudes and practices (KAP) survey, a CHV digital needs assessment and the DCE reported here.

The objective of this study is to answer the following research questions: (i) what are the most preferred interventions that promote healthier eating and physical activity among B40 communities in urban Kuala Lumpur? and (ii) what is the strength of their preferences for these interventions?

## METHODOLOGY

### Study design and setting

The DCE was conducted among residents of low-cost apartments in urban Kuala Lumpur. Kuala Lumpur is administratively divided into *Kawasan Rukun Tetangga* (KRT). Of the 307 KRTs in Kuala Lumpur, 48 are classified under People’s Housing Project (PPR) and Public Housing (PA), which are often used as a proxy for the B40 population. The DCE was conducted in the three intervention KRTs of the BHP: (i) PPR Pekan Kepong Setia, (ii) PA Sri Negeri Sembilan at Kepong district and (iii) PPR Seri Kota at Cheras district (Kataria *et al.*, 2020).

To be eligible to participate, participants had to be 18 years of age or older, a resident of the selected PPR or PA, and willing and able to provide informed consent. The minimum required sample size for the DCE was calculated using Orme’s sample size rule (Orme, 2005). The minimum required sample size was 150 respondents. Our target sample size was 480 respondents per KRT, stratified by age and sex based on the state-level population.

The DCE protocol was reviewed and approved by the Medical Research and Ethics Committee (MREC) of the Ministry of Health Malaysia (MOH) on 7 July 2020 (Approval number: KKM/NIHSEC/P20-1156(12)).

### Survey design

DCEs quantify respondent preferences for interventions by asking individuals to make choices between different hypothetical alternatives (Mangham *et al.*, 2009). In this study, these alternatives are combinations of potential obesity-prevention interventions that could be implemented in the community. The initial list of potential interventions to be included as attributes in the survey was drawn from a literature review of effective community-based obesity-prevention interventions. This list was shared with members of the KRT leadership committees, Ministry of Health officials, and district health officials for feedback on which interventions were most acceptable to the KRT context. The final selection was also informed by the results of a mapping of the social and environmental context of the intervention KRTs, including the local stores, food vendors, and built space for physical activity and a mixed-methods survey to assess the impact of COVID-19 Movement Control Orders on community member health behaviours and NCD risk factors conducted by BHP Malaysia (Lim *et al.*, 2022). Based on these sources, the initial list was narrowed to the 14 final interventions (Table 1).

**Table 1:** Selected interventions and levels

Interventions/attributes	Levels
1.Changes to reduce salt in foods prepared at vendors and restaurants	a.No changes to salt in foods prepared at vendors and restaurants b.Reduced salt in foods prepared at vendors and restaurants
2.Changes to reduce oil in foods prepared at vendors and restaurants	a.No changes to oil in foods prepared at vendors and restaurants b.Reduced oil in foods prepared at vendors and restaurants
3.Changes to reduce sugar in foods prepared at vendors and restaurants	a.No changes to sugar in foods prepared at vendors and restaurants b.Reduced sugar in foods prepared at vendors and restaurants
4.Changes to increase vegetables in foods prepared at vendors and restaurants	a.No changes to vegetables in foods prepared at vendors and restaurants b.Increased vegetables in foods prepared at vendors and restaurants
5.Changes to labelling to promote healthier options at food outlets	a.No changes in labelling at food outlets b.Labelling at food outlets to indicate healthier food options
6.Changes in the price of fruits at neighbourhood grocery stores	a.No change in price of fruits at neighbourhood grocery stores b.5% decrease in price of fruits at neighbourhood grocery stores c.10% decrease in price of fruits at neighbourhood grocery stores
7.Changes in the price of vegetables at neighbourhood grocery stores	a.No change in price of vegetables at neighbourhood grocery stores b.5% decrease in price of vegetables at neighbourhood grocery stores c.10% decrease in price of vegetables at neighbourhood grocery stores
8.Earn rewards points for completing online health, diet and physical activity education	a.No rewards points available for completing online health, diet and physical activity education. b.Earn rewards points redeemable for RM 2 voucher for completing online health, diet and physical activity education c.Earn rewards points redeemable for RM 5 voucher for completing online health, diet and physical activity education
9.Earn rewards points for attending events such as health education workshops, cooking classes and physical activity groups	a.No rewards points available for attending in-person health promotion events b.Earn rewards points redeemable for RM 2 voucher for attending in-person health promotion events c.Earn rewards points redeemable for RM 5 voucher for attending in-person health promotion events
10.Changes to labelling of products in neighbourhood grocery stores	a.Make no changes to labelling of food in neighbourhood grocery stores b.Place signs inside your neighbourhood grocery store to show which foods are healthier choices c.Place signs inside your neighbourhood grocery stores to which foods are unhealthy choices
11.Changes to placement of healthier food options in neighbourhood grocery stores to improve visibility and easy access	a.No changes to placement of healthier food options in neighbourhood grocery stores b.Healthier food options to be placed at the eye level and nearest to payment counter
12.Cooking classes to demonstrate affordable, healthy recipes for home cooking	a.Cooking classes not offered b.Cooking classes to demonstrate affordable, healthy recipes for home cooking offered through online videos c.Cooking classes to demonstrate affordable, healthy recipes for home cooking offered in person
13.Organized physical activities for community members	a.No organized physical activities are offered b.Online resources for physical activity through work out plans or videos are offered c.In person physical activity classes and clubs are offered
14.Health educational resources to improve knowledge about common health problems and risk factors, strategies for healthy eating on a budget, and/or ideas of being physically active at home or in the community	a.Health education resources are not offered b.Educational resources about common health problems, healthy eating and physical activity are offered through a mobile app c.Educational workshops about common health problems, healthy eating and physical activity are offered in person

The core of a DCE survey is the choice questions. This survey used a partial-profile design, with each hypothetical alternative including a combination of 3 interventions selected from the list of 14 possible interventions (Table 1). This approach simplified the survey and reduced the response burden. Each participant responded to a different series of choice questions, which vary in terms of the interventions and levels of the interventions included. For example, price interventions included decreases in the price of fruits by 0%, 5% and 10%. We used Sawtooth Choice-Based Conjoint Software to generate an efficient partial-profile design that chose an optimal sample of all hypothetical intervention combinations (Zwerina *et al.*, 1996; Kanninen, 2002).

**Fig. 1:**
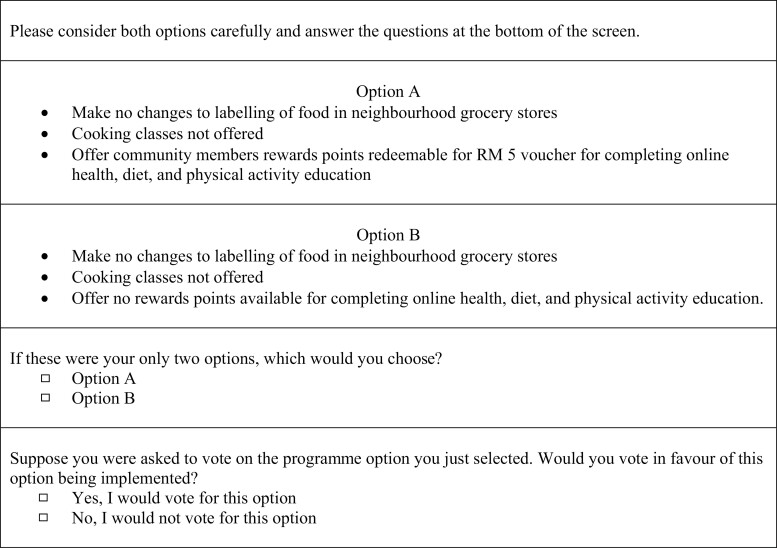
Sample choice task question.

Each choice question had two parts. First, the respondent chose between two hypothetical sets of interventions (Option A and Option B). Next, they were asked if they would vote for this option to be implemented. By combining respondents’ answers to these two questions, their choice is really being made between three alternatives: Option A, Option B or Neither. An example of this procedure is provided in Figure 1.

The survey included 12 total choice questions. Ten of the 12 questions were used in the primary analysis of the survey results, and the 2 remaining questions were used to assess the validity of these results. These two questions included a logic test question and a holdout task. In the logic test, one option is intended to be clearly preferable to the other (Figure 1). If respondents choose the non-preferred option, this result indicates that they may not sufficiently understand the choice questions. The holdout task is an additional choice question that is used to test how accurately the model predicts responses. If the predictions match the actual responses to this question, the preferences quantified by the statistical model are considered reliable (Orme, 2015). Respondents also provided demographic information.

### Pilot testing

Before administering the survey, we pilot tested the DCE questionnaire through 15 cognitive interviews. Fifteen community members, representing all 3 participating KRTs, completed the questionnaire and responded to questions on the length, clarity and their comprehension of the questionnaire. Revisions were made to address the feedback on the clarity and length of the survey, and the revised questionnaire was re-tested with six community members, who reported improved experience and understanding.

### Data collection

All surveys were completed in person by trained interviewers using the Voxco survey platform in December 2020. All interviewers underwent thorough training on the study protocol, data collection methods, study questionnaire, data confidentiality and COVID-19 safety. All respondents provided written informed consent. The questionnaire was developed in English, and then translated into Malay and Mandarin, with participants able to select their preferred language. Interviewers read all questions to participants, who gave oral responses. Participants who were able to read also had the option to view the choice questions in writing. Data were stored in the RTI Enhanced Security Network. Survey responses were cross-referenced to identify duplicate respondents. Where identified, only the participant’s first response was retained for analysis.

### Data analysis

Participants’ responses to the choice questions were used to determine which health intervention the average respondent most preferred. In line with the best practices, these data were used to generate a random utility maximization (RUM) model (Bridges et al., 2011). We assume that respondents will choose the option that gives them the highest expected utility; so this model allows us to estimate the utility gain associated with each intervention. We defined the utility a person receives from option *j* on choice task *t* by


$$\begin{array}{l} u_{jt}=v_{jt}\left(X_{jt}\right)+\varepsilon_{jt},\;j=\;0,\;1,\;2,\;t\;=\;1,\ldots .,10, \end{array}$$ (1)

where *v*_*j*_ is the deterministic (observable) component of utility that depends on the attribute levels that compose option *j* in choice task *t* (represented as the vector **X**_*jt*_), and *ε*_*j*_ is a random error that represents the component of utility that is unobservable to the researcher. Given this framework, the probability that option *j* will be chosen over option *k* is $\mathrm{Pr}\left(u_{j}>u_{k}\right)=\mathrm{Pr}\left(\varepsilon_{k}-\varepsilon_{j}<v_{j}-v_{k}\right)$. The random error terms were assumed to be independently and identically distributed extreme value.

The RUM model was estimated using a conditional logit model in Stata 16. We estimated this model for the full sample and then separately for each of the three KRTs. We assumed that a respondent’s utility (or satisfaction) from implementing an intervention was specified as linear in intervention attributes, and the utility of not implementing an intervention was modelled as an alternative specific constant. In addition, following the best practice methods, we treated all variables as an additive in categorical attribute levels and coded them for effects for improved statistical properties.

The coefficients from the model can be interpreted as satisfaction scores for the average respondent, with larger scores indicating greater satisfaction. Using the satisfaction scores, we then calculate importance scores to rank which interventions would result in the largest change in respondent satisfaction.

We analysed the validity check questions to assess our confidence that the estimated rankings accurately reflect respondent preferences. To analyse the results of the logic test, we calculated the percentage of respondents that passed the test. To analyse the holdout task, we used the satisfaction scores to estimate the probability that respondents will choose Option A, Option B or Neither. The predicted proportions of respondents choosing each option were then compared to respondent’s actual responses using a *z*-score test.

Finally, we used a Pearson’s Chi-square test of independence of two categorical variables to test for differences in the distribution of responses across KRTs.

## RESULTS

### Respondent characteristics

A total of 1453 participants responded to the DCE survey (Table 2). Three-quarters (76%) of the respondents were 50 years or younger; 77% had completed the equivalent of secondary school education or higher; 54% were Malay ethnicity and 52% were female. Almost all respondents (94%) had a total monthly household income of RM 5000 or less (~USD 1180). Respondent characteristics by KRT are presented in Table S1.

**Table 2:** Respondent demographics (*n* = 1453)

	*n* (%)
Respondent sex	
Male	697 (48.2)
Female	748 (51.8)
Did not answer/ refused to answer	8 (0.6)
Respondent age	
30 years old or younger	537 (37.0)
31–50 years	571 (39.3)
Older than 50 years	345 (23.7)
Respondent education	
Primary school or below	120 (8.3)
Lower secondary school ^a^	205 (14.1)
Higher secondary school ^b^	924 (63.6)
Pre-university or above	196 (13.5)
Did not answer/ Refused to Answer	8 (0.6)
Monthly household income (RM)	
<3000	889 (61.2)
3000–4999	483 (33.2)
≥5000	47 (3.2)
Refused to answer	34 (2.3)
Respondent ethnicity	
Malay	780 (53.7)
Chinese	339 (23.3)
Indian	322 (22.2)
Other	3 (0.2)
Did not answer/ refused to answer	9 (0.6)

RM, Ringgit Malaysia.

^a^ Completion of Lower Secondary Assessment Examination or equivalent in secondary school in Malaysia, indicating about 9 years of formal education.

^b^ Completion of Malaysian Higher School Certificate or equivalent in secondary school in Malaysia, indicating about 11 years of formal education.

### Satisfaction scores

Participants indicated statistically significant increases in satisfaction from all interventions, except for the health educational resources (Table 3). These satisfaction scores quantify preferences for the average respondent for the given group, with a higher score indicating that the intervention level yields more satisfaction compared to a level with a lower score.

**Table 3:** Estimated satisfaction scores by attribute and relative importance of each intervention (*n* = 1453)

Interventions	Satisfaction scores		Importance scores		
	Mean satisfaction score	Standard error	Potential impact on satisfaction	Relative importance score	Rank
Changes in the price of fruits at neighbourhood grocery stores			0.73	16.79	1
No change	−0.36***	0.03			
5% decrease	−0.02***	0.03			
10% decrease	0.38***	0.03			
Changes in the price of vegetables at neighbourhood grocery stores			0.65	14.89	2
No change	−0.34***	0.03			
5% decrease	0.02***	0.03			
10% decrease	0.31***	0.03			
Earn rewards points for completing online health, diet and physical activity education.			0.39	8.86	3
No rewards	−0.21***	0.03			
RM 2 voucher	0.04***	0.03			
RM 5 voucher	0.18***	0.03			
Changes to reduce sugar in foods prepared at vendors and restaurants			0.34	7.85	4
No changes	−0.17***	0.02			
Reduced sugar	0.17***	0.02			
Changes to reduce oil in foods prepared at vendors and restaurants			0.30	6.89	5
No changes	−0.15***	0.02			
Reduced oil	0.15***	0.02			
Changes to reduce salt in foods prepared at vendors and restaurants			0.30	6.80	6
No changes	−0.15***	0.02			
Reduced salt	0.15***	0.02			
Earn rewards points for attending in-person health promotion events.			0.29	6.59	7
No rewards	−0.16***	0.03			
RM 2 voucher	0.03***	0.03			
RM 5 voucher	0.13**	0.03			
Organized physical activities for community members			0.25	5.70	8
No organized physical activities	−0.17***	0.03			
Online resources	0.1***	0.03			
In person physical activity classes and clubs	0.08	0.03			
Changes to increase vegetables in foods prepared at vendors and restaurants			0.25	5.70	9
No changes	−0.12***	0.02			
Increased vegetables	0.12***	0.02			
Changes to labelling to promote healthier options at food outlets.			0.22	5.04	10
No changes	−0.11***	0.02			
Labelling at food outlets to indicate healthier food options	0.11***	0.02			
Changes to placement of healthier food options in neighbourhood grocery stores to improve visibility and easy access			0.22	4.92	11
No changes	−0.11***	0.02			
Healthier food options at the eye level and nearest to payment counter	0.11***	0.02			
Cooking classes to demonstrate affordable, healthy recipes for home cooking			0.19	4.33	12
No cooking classes	−0.15***	0.03			
Cooking classes through online videos	0.12***	0.03			
Cooking classes in person	0.03	0.03			
Health educational resources to improve knowledge about common health problems and risk factors, strategies for healthy eating on a budget, and/or ideas of being physically active at home or in the community.			0.15	3.49	13
No resources	−0.07	0.03			
Educational resources through a mobile app	−0.01	0.03			
Educational workshops in person	0.08*	0.03			
Changes to labelling of products in neighbourhood grocery stores			0.09	2.17	14
No changes	−0.08***	0.03			
Visual labels to indicate healthier food options	0.06***	0.03			
Visual labels to indicate high levels of salt, oil, or sugar	0.02	0.03			
Total			4.37	100.00	

Note: Standard errors on omitted coefficients calculated using delta method. Adjacent attribute level statistical significance tests were conducted using the delta method.

***p < 0.01, **p < 0.05, *p < 0.10.

### Importance scores

The importance scores indicate which interventions would result in the largest change in respondent satisfaction (Table 3). The ‘potential impact on satisfaction’ scores indicate how much respondent satisfaction will increase if you were to change each intervention from its least preferred level to its most preferred level. The ‘relative importance’ scores indicate what proportion of the respondent’s total satisfaction is attributable to this intervention.

Across the KRTs, the interventions to reduce the price of fruit and vegetables and to offer reward points in return for completing online health education modules were most important. Next, the most important were the interventions to adjust cooking practices at food vendors and restaurants. Generally, the interventions related to labelling, placement of healthy foods, and educational resources and activities were rated as less important.

The intervention ranking and associated satisfaction and importance scores by KRT are presented in Tables S2 and S3.

### Validity test results

We assessed the validity of our analytical results using the holdout task and logic test. For holdout task, the choice predicted by the conditional logit results differed from actual choices by an average of two percentage points (Table 4). The difference between predicted and actual choices falls outside the 95% CI for Option A, indicating that they are statistically significant. For the logic test, we found that 35% of all respondents selected the less preferred option, indicating that they failed the logic test. The predicted and actual responses to the holdout task question by KRT are presented in Table S4.

**Table 4:** Predicted and actual responses to holdout task question (*n* = 1453)

	Predicted percentage of respondents choosing each option in holdout task	Actual percentage of respondents choosing each option in holdout task
	% [95% CI]	
Option A	48% [43%–51%]	42%
Option B	44% [41%–49%]	49%
Neither	9% [8%–9%]	9%

## DISCUSSION

This study contributes to our understanding of community preferences for obesity-control interventions among the urban B40 in Kuala Lumpur. Our findings indicate that priority interventions are those that (i) reduce prices of fruit and vegetables; (ii) support healthier cooking practices at restaurants and food vendors and (iii) offer reward incentives for completing online educational activities.

The popularity of interventions related to price is consistent with the prior finding that food prices affect food choices, especially among people with lower socio-economic status (Pondor *et al.*, 2017). Affordability has been previously highlighted as one of the barriers to fruit consumption among young urban Malaysian adults (Abdul-Hakim *et al.*, 2018). Similarly, in the baseline KAP survey conducted by BHP Malaysia, price was the most commonly reported factor affecting food purchasing decisions (Eng *et al.*, 2022). As such, strategies to reduce the prices of healthy foods have the potential to improve their affordability and accessibility.

Changes to the out-of-home food environment were also a high priority across KRTs. Eating out is a common practice in Kuala Lumpur given that long working hours limit the time available to prepare meals. In the BHP Malaysia KAP survey, 46.3% of respondents indicated that they ate out at least once per week (Eng et al., 2022). Foods prepared outside of the home tend to be more energy-dense, nutrient-poor, and high in salt, oil and sugar (Azizan *et al.*, 2018; Balasubramanian et al., 2020; Goh *et al.*, 2020; Goh & Choong, 2020). Changes to the out-of-home food environment would, therefore, be potentially beneficial to reduce the consumption of less healthy food (Neckerman, 2014). Such an intervention is a prime example of a nudge that could alter the decision environment for community members without requiring any individual changes to the behaviour of eating out.

Respondents also preferred interventions that offered reward points for completing online educational activities, which could be redeemed for cash vouchers to buy healthier food items at neighbourhood shops. Interest in these rewards may relate to the financial concerns of the communities. Previous studies have established the effectiveness of using incentives to encourage participation in health promotion interventions (Marteau *et al.*, 2009). The preference for online activities over in-person activities is notable, and might be due to a perception that online resources are more flexible and accessible. Given the timing of this study (December 2020), this preference may also reflect concerns about the risk of COVID-19 transmission through in-person gatherings.

Community members did not prefer several interventions, including changes to product placement or product labelling. It is important to note that DCEs identify preferences for interventions, and not all effective interventions will be appealing or preferable to individuals. While community preferences should inform interventions design, there is also an important role for interventions that may be less popular but are effective in nudging individuals towards healthier choices, such as taxes on tobacco products or sugar-sweetened beverages (Chaloupka *et al.*, 2012; Hagmann *et al.*, 2018). For this reason, the findings of this DCE were just one component of BHP Malaysia’s intervention selection approach.

Informed by these findings and other formative research activities (Eng *et al.*, 2022; Lim *et al.*, 2022), BHP Malaysia proposed a suite of locally tailored, evidence-based, community-informed interventions for NCD and obesity prevention with three objectives:

- Improving access to, promoting and incentivizing healthy eating in partnership with local businesses;
- Building knowledge, capacity, and skills for healthier eating and NCD prevention among CHVs and community members; and
- Providing and promoting opportunities to engage in physical activity through attractive and accessible virtual and community programming.

## LIMITATIONS

The DCE used an innovative and rigorous methodology to quantify the preferences of community members related to potential obesity-prevention interventions. While we believe that this study offers genuine insights into participant preferences, there are several important limitations to consider.

DCEs, like all stated preference methods, have been critiqued for potentially being susceptible to hypothetical bias, in which participants report theoretical preferences not consistent with their choices in reality (Viney *et al.*, 2002). Our sample was recruited to be representative of the state-level population in terms of age and gender, but our estimates may be subject to non-response bias. Further, a total of 50 duplicate responses were identified among the participants surveyed. Repeat responses were excluded from the final dataset, and their exclusion did not significantly change the results.

The results of the validity test questions merit further consideration. The holdout task analysis indicates that there are slight statistically significant differences between participants’ predicted and actual responses. These differences are notable, but do not invalidate our findings. While the holdout task analysis is a recommended validity test, the aim of the DCE analysis is not to predict responses, but to identify which attributes are most important to decision-making. As a result, even if the model does not always accurately predict participants’ choices when faced with two similar options, this does not indicate that the model is incorrectly estimating the importance of the included attributes.

A relatively large percentage of respondents (35%) failed a logic test. In a 2019 systematic review of DCEs, approximately one-quarter of studies reported a failure rate greater than 27% for the logic test (Johnson *et al.*, 2019). We believe this failure rate was due to two factors. First, the format in which the DCE was administered might have contributed to a greater likelihood in respondents making random mistakes when answering survey questions. Participants who were not able to read were reliant on the interviewer delivering the questionnaire orally, which might have made a comparison of the different options in the choice tasks challenging. However, the results of the analysis are similar when excluding respondents that failed the logic test, suggesting that the failure rate did not bias the results. This random error could potentially help explain why the RUM did not accurately predict all of the holdout task responses.

Second, given the resulting model estimates, we can see that the anticipated satisfaction associated with the ‘better’ option in the logic test was not much greater than that of the ‘worse’ option. As a result, random error might have played a larger role in decision-making for this question than anticipated. Future studies can utilize the satisfaction scores we estimated to better construct a logic test in which there is a more clearly preferable option.

## CONCLUSIONS

The DCE enabled the clear articulation of community member preferences for interventions to improve access to healthier food options and physical activities in low-income communities in Kuala Lumpur. The study identified that the most preferred interventions were those to improve the affordability and availability of healthier foods and offer financial incentives for completing online health educational modules. Making healthy choices more accessible, affordable and attractive to the community came out strongly in the elicited preferences, highlighting the salience of nudges and environmental interventions that make the healthy choice the easy choice. These findings can help policy-makers and public health professionals responsively tailor future interventions for obesity prevention. This study also demonstrated the feasibility and potential value of using a DCE to generate robust insights into community priorities for health promotion. Our experiences generated learnings on the application of partial-profile methodologies in the context of a low-resource setting, particularly in relation to the design of validity test questions. These learnings assist successful application of this methodology in future studies. The findings of this study were used to inform the design of the implementation strategy of the BHP Malaysia community health promotion and obesity-prevention programme.

## Supplementary Material

daac156_suppl_Supplementary_Material
