# Supplementary material for: Community priorities for obesity prevention among low-income adults in Kuala Lumpur: a discrete choice experiment
Source: Health Promot Int. 2022 Nov 11;37(6):daac156. doi: 10.1093/heapro/daac156 (PMC9651037; doi:10.1093/heapro/daac156)
Supplement: daac156_suppl_Supplementary_Material [file daac156_suppl_supplementary_material.docx]

**Community priorities for obesity prevention among low-income adults in Kuala Lumpur: A discrete choice experiment**

Erica Kocher, Dallas Wood, Shiang-Cheng Lim, Angie Jackson-Morris, Ishu Kataria, Carrie Ngongo, Zhi Sham, Arunah Chandran, Rachel Nugent, and Feisul Idzwan Mustapha

*Supplementary Materials*

[Table S1. Respondent demographics, by KRT 2](#_Toc113544192)

[Table S2: Satisfaction Score by Attribute as Estimated Using a Conditional Logit Model, by KRT 3](#_Toc113544193)

[Table S3: Relative Importance of Each Intervention, by KRT 5](#_Toc113544194)

[Table S4. Evaluating Predicted Responses to Holdout Task Question (95% Confidence Intervals) 6](#_Toc113544195)

### Table S1. Respondent demographics, by KRT

|  | Total (n=1,453)  n (%) | PPR Pekan Kepong  (n=491)  n (%) | | PA Sri Negeri Sembilan (n=489)  n (%) | | PPR Seri Kota  (n=473)  n (%) | | | | P-value* |
| --- | --- | --- | --- | --- | --- | --- | --- | --- | --- | --- |
| **Respondent Sex 0.787** | | | | | | | | | | |
| Male | 697 (48.2) | | 234 (48.3) | 230 (47.1) | 233 (49.4) | | | |  | |
| Female | 748 (51.8) | | 251 (51.8) | 258 (52.9) | 239 (50.6) | | | |  | |
| Did not answer / Refused to Answer | 8 (0.6) | | 6 (1.2) | 1 (0.2) | 1 (0.2) | | | |  | |
| **Respondent Age 0.948** | | | | | | | | | | |
| 30 years old or younger | 537 (37.0) | | 187 (38.1) | 174 (35.6) | 176 (37.2) | | | |  | |
| 31–50 years | 571 (39.3) | | 191 (38.9) | 196 (40.1) | 184 (38.9) | | | |  | |
| Older than 50 years | 345 (23.7) | | 113 (23.0) | 119 (24.3) | 113 (23.9) | | | |  | |
| **Respondent Education** | | | | | | |  | **<0.001** | | |
| Primary school or below | 120 (8.3) | | 41 (8.4) | 43 (8.8) | 36 (7.6) | | | |  | |
| Lower secondary school ^a^ | 205 (14.1) | | 86 (17.5) | 78 (16.0) | 41 (8.7) | | | |  | |
| Higher secondary school ^b^ | 924 (63.6) | | 329 (67.0) | 310 (63.4) | 285 (60.3) | | | |  | |
| Pre-university or above | 196 (13.5) | | 29 (5.9) | 57 (11.7) | 110 (23.3) | | | |  | |
| Did not answer / Refused to Answer | 8 (0.6) | | 6 (1.2) | 1 (0.2) | 1 (0.2) | | | |  | |
| **Monthly Household Income (RM)** | | | | | | |  | **<0.001** | | |
| <3,000 | 889 (61.2) | | 323 (65.8) | 313 (64.0) | 253 (53.5) | | | |  | |
| 3000-4999 | 483 (33.2) | | 129 (26.3) | 153 (31.3) | 201 (42.5) | | | |  | |
| ≥5000 | 47 (3.2) | | 20 (4.1) | 12 (2.5) | 15 (3.2) | | | |  | |
| Refused to answer | 34 (2.3) | | 19 (3.9) | 11 (2.3) | 4 (0.9) | | | |  | |
| **Respondent Ethnicity** | | | | | | |  | **<0.001** | | |
| Malay | 780 (53.7) | | 238 (48.5) | 215 (44.0) | 327 (69.1) | | | |  | |
| Chinese | 339 (23.3) | | 190 (38.7) | 83 (17.0) | 66 (14.0) | | | |  | |
| Indian | 322 (22.2) | | 55 (11.2) | 188 (38.5) | 79 (16.7) | | | |  | |
| Other | 3 (0.2) | | 1 (0.2) | 2 (0.4) | 0 (0.0) | | | |  | |
| Did not answer / Refused to Answer | 9 (0.6) | | 7 (1.4) | 1 (0.2) | 1 (0.2) | | | |  | |

*RM, Ringgit Malaysia*

*^a^ Completion of Lower Secondary Assessment Examination or equivalent in secondary school in Malaysia, indicating about 9 years of formal education.*

*^b^ Completion of Malaysian Higher School Certificate or equivalent in secondary school in Malaysia, indicating about 11 years of formal education.*

**Test for differences in responses across subsamples using chi-square test.*

### Table S2: Satisfaction Score by Attribute as Estimated Using a Conditional Logit Model, by KRT

|  | **Total**  **(n=1,453)** | | **PPR Pekan Kepong**  **(n= 491)** | | **PA Sri Negeri Sembilan (n= 489)** | | **PPR Seri Kota**  **(n= 473)** | |
| --- | --- | --- | --- | --- | --- | --- | --- | --- |
| **Attributes** | **Mean Satisfaction Score** | **Standard Error** | **Mean Satisfaction Score** | **Standard Error** | **Mean Satisfaction Score** | **Standard Error** | **Mean Satisfaction Score** | **Standard Error** |
| 1. Changes to reduce salt in foods prepared at vendors and restaurants |  |  |  |  |  |  |  |  |
| No changes in foods prepared at vendors and restaurants | -0.15*** | 0.02 | -0.14*** | 0.03 | -0.14*** | 0.03 | -0.18*** | 0.03 |
| Reduced salt in foods prepared at vendors and restaurants | 0.15*** | 0.02 | 0.14*** | 0.03 | 0.14*** | 0.03 | 0.18*** | 0.03 |
| 2. Changes to reduce oil in foods prepared at vendors and restaurants |  |  |  |  |  |  |  |  |
| No changes in foods prepared at vendors and restaurants | -0.15*** | 0.02 | -0.08** | 0.03 | -0.18*** | 0.03 | -0.19*** | 0.03 |
| Reduced oil in foods prepared at vendors and restaurants | 0.15*** | 0.02 | 0.08** | 0.03 | 0.18*** | 0.03 | 0.19*** | 0.03 |
| 3. Changes to reduce sugar in foods prepared at vendors and restaurants |  |  |  |  |  |  |  |  |
| No changes in foods prepared at vendors and restaurants | -0.17*** | 0.02 | -0.13*** | 0.03 | -0.14*** | 0.03 | -0.25*** | 0.03 |
| Reduced sugar in foods prepared at vendors and restaurants | 0.17*** | 0.02 | 0.13*** | 0.03 | 0.14*** | 0.03 | 0.25*** | 0.03 |
| 4. Changes to increase vegetables in foods prepared at vendors and restaurants |  |  |  |  |  |  |  |  |
| No changes in foods prepared at vendors and restaurants | -0.12*** | 0.02 | -0.09*** | 0.03 | -0.09*** | 0.03 | -0.19*** | 0.03 |
| Increased vegetables in foods prepared at vendors and restaurants | 0.12*** | 0.02 | 0.09*** | 0.03 | 0.09*** | 0.03 | 0.19*** | 0.03 |
| 5. Changes to labelling to promote healthier options at food outlets. |  |  |  |  |  |  |  |  |
| No changes in labelling at food outlets | -0.11*** | 0.02 | -0.09*** | 0.03 | -0.11*** | 0.03 | -0.13*** | 0.03 |
| Labelling at food outlets to indicate healthier food options | 0.11*** | 0.02 | 0.09*** | 0.03 | 0.11*** | 0.03 | 0.13*** | 0.03 |
| 6. Changes in the price of fruits at neighbourhood grocery stores |  |  |  |  |  |  |  |  |
| No change in price of fruits at neighbourhood grocery stores | -0.36*** | 0.03 | -0.26*** | 0.05 | -0.21** | 0.06 | -0.63*** | 0.06 |
| 5% decrease in price of fruits at neighbourhood grocery stores | -0.02*** | 0.03 | 0*** | 0.05 | -0.01** | 0.05 | -0.05*** | 0.06 |
| 10% decrease in price of fruits at neighbourhood grocery stores | 0.38*** | 0.03 | 0.26*** | 0.05 | 0.22** | 0.05 | 0.68*** | 0.06 |
| 7. Changes in the price of vegetables at neighbourhood grocery stores |  |  |  |  |  |  |  |  |
| No change in price of vegetables at neighbourhood grocery stores | -0.34*** | 0.03 | -0.25*** | 0.05 | -0.29*** | 0.06 | -0.49*** | 0.06 |
| 5% decrease in price of vegetables at neighbourhood grocery stores | 0.02*** | 0.03 | 0.02*** | 0.05 | 0.02*** | 0.05 | 0.03*** | 0.05 |
| 10% decrease in price of vegetables at neighbourhood grocery stores | 0.31*** | 0.03 | 0.23** | 0.05 | 0.27*** | 0.05 | 0.46*** | 0.06 |
| 8. Earn rewards points for completing online health, diet, and physical activity education. |  |  |  |  |  |  |  |  |
| No rewards points available | -0.21*** | 0.03 | -0.15* | 0.05 | -0.28*** | 0.05 | -0.21* | 0.06 |
| Earn rewards points redeemable for RM 2 voucher for completing online health, diet, and physical activity education. | 0.04*** | 0.03 | 0.03* | 0.05 | 0.1*** | 0.05 | -0.03* | 0.05 |
| Earn rewards points redeemable for RM 5 voucher for completing online health, diet, and physical activity education. | 0.18*** | 0.03 | 0.12 | 0.05 | 0.18 | 0.05 | 0.25*** | 0.05 |
| 9. Earn rewards points for attending in-person health promotion events. |  |  |  |  |  |  |  |  |
| No rewards points available | -0.16*** | 0.03 | -0.13 | 0.05 | -0.14** | 0.05 | -0.21** | 0.06 |
| Earn rewards points redeemable for RM 2 voucher for attending in-person health promotion events. | 0.03*** | 0.03 | 0 | 0.05 | 0.05** | 0.05 | 0.03** | 0.06 |
| Earn rewards points redeemable for RM 5 voucher for attending in-person health promotion event. | 0.13** | 0.03 | 0.13 | 0.05 | 0.09 | 0.05 | 0.19* | 0.05 |
| 10. Changes to labelling of products in neighbourhood grocery stores |  |  |  |  |  |  |  |  |
| No changes to labelling of food in neighbourhood grocery stores | -0.08*** | 0.03 | -0.04 | 0.05 | -0.14** | 0.05 | -0.06 | 0.05 |
| Visual labels on foods in neighbourhood grocery stores to indicate healthier food options | 0.06*** | 0.03 | 0.07 | 0.05 | 0.07** | 0.05 | 0.05 | 0.05 |
| Visual labels on foods in neighbourhood grocery stores to indicate high levels of salt, oil, or sugar | 0.02 | 0.03 | -0.03 | 0.05 | 0.08 | 0.05 | 0.01 | 0.05 |
| 11. Changes to placement of healthier food options in neighbourhood grocery stores to improve visibility and easy access |  |  |  |  |  |  |  |  |
| No changes to placement of healthier food options in neighbourhood grocery stores | -0.11*** | 0.02 | -0.09*** | 0.03 | -0.09*** | 0.03 | -0.14*** | 0.03 |
| Healthier food options to be placed at the eye level and nearest to payment counter | 0.11*** | 0.02 | 0.09*** | 0.03 | 0.09*** | 0.03 | 0.14*** | 0.03 |
| 12. Cooking classes to demonstrate affordable, healthy recipes for home cooking |  |  |  |  |  |  |  |  |
| Cooking classes not offered | -0.15*** | 0.03 | -0.21*** | 0.05 | -0.07 | 0.05 | -0.19*** | 0.06 |
| Cooking classes to demonstrate affordable, healthy recipes for home cooking offered through online videos | 0.12*** | 0.03 | 0.17*** | 0.05 | 0.02 | 0.05 | 0.17*** | 0.05 |
| Cooking classes to demonstrate affordable, healthy recipes for home cooking offered in person | 0.03 | 0.03 | 0.04 | 0.05 | 0.04 | 0.05 | 0.03 | 0.05 |
| 13. Organized physical activities for community members |  |  |  |  |  |  |  |  |
| No organized physical activities | -0.17*** | 0.03 | -0.19*** | 0.05 | -0.06 | 0.05 | -0.27*** | 0.06 |
| Online resources for physical activity through work out plans or videos | 0.1*** | 0.03 | 0.1*** | 0.05 | 0.01 | 0.05 | 0.19*** | 0.05 |
| In person physical activity classes and clubs | 0.08 | 0.03 | 0.09 | 0.05 | 0.05 | 0.05 | 0.08 | 0.06 |
| 14.   Health educational resources to improve knowledge about common health problems and risk factors, strategies for healthy eating on a budget, and/or ideas of being physically active at home or in the community. |  |  |  |  |  |  |  |  |
| Resources are not offered | -0.07 | 0.03 | -0.14 | 0.05 | 0 | 0.05 | -0.07 | 0.06 |
| Educational resources about common health problems, healthy eating, and physical activity are offered through a mobile app | -0.01 | 0.03 | 0.01 | 0.05 | -0.05 | 0.05 | 0.01 | 0.05 |
| Educational workshops about common health problems, healthy eating, and physical activity are offered in person | 0.08* | 0.03 | 0.13 | 0.05 | 0.05 | 0.05 | 0.06 | 0.05 |

*Note: Standard errors on omitted coefficients calculated using delta method. Adjacent attribute level statistical significance tests were conducted using the delta method.*

## Table S3: Relative Importance of Each Intervention, by KRT

|  | **Total  (n= 1,453)** | | | **PPR Pekan Kepong  (n= 491)** | | | **PA Sri Negeri Sembilan  (n= 489)** | | | **PPR Seri Kota  (n= 473)** | | |
| --- | --- | --- | --- | --- | --- | --- | --- | --- | --- | --- | --- | --- |
|  | **Potential Impact on Satisfaction** | **Relative Importance Score** | **Rank** | **Potential Impact on Satisfaction** | **Relative Importance Score** | **Rank** | **Potential Impact on Satisfaction** | **Relative Importance Score** | **Rank** | **Potential Impact on Satisfaction** | **Relative Importance Score** | **Rank** |
| Changes in the price of fruits at neighbourhood grocery stores | 0.73 | 16.79 | 1 | 0.52 | 14.52 | 1 | 0.43 | 11.64 | 3 | 1.31 | 21.60 | 1 |
| Changes in the price of vegetables at neighbourhood grocery stores | 0.65 | 14.89 | 2 | 0.47 | 13.18 | 2 | 0.56 | 15.37 | 1 | 0.95 | 15.71 | 2 |
| Earn rewards points for completing online health, diet, and physical activity education. | 0.39 | 8.86 | 3 | 0.27 | 7.56 | 5 | 0.46 | 12.53 | 2 | 0.46 | 7.57 | 4 |
| Changes to reduce sugar in foods prepared at vendors and restaurants | 0.34 | 7.85 | 4 | 0.26 | 7.32 | 7 | 0.28 | 7.51 | 6 | 0.50 | 8.32 | 3 |
| Changes to reduce oil in foods prepared at vendors and restaurants | 0.30 | 6.89 | 5 | 0.27 | 7.67 | 4 | 0.29 | 7.82 | 5 | 0.35 | 5.86 | 8 |
| Changes to reduce salt in foods prepared at vendors and restaurants | 0.30 | 6.80 | 6 | 0.15 | 4.20 | 13 | 0.36 | 9.86 | 4 | 0.39 | 6.39 | 6 |
| Earn rewards points for attending in-person health promotion events. | 0.29 | 6.59 | 7 | 0.26 | 7.26 | 8 | 0.22 | 6.09 | 8 | 0.40 | 6.56 | 5 |
| Organized physical activities for community members | 0.25 | 5.70 | 8 | 0.29 | 7.96 | 3 | 0.11 | 2.94 | 13 | 0.35 | 5.80 | 9 |
| Changes to increase vegetables in foods prepared at vendors and restaurants | 0.25 | 5.70 | 9 | 0.18 | 5.13 | 12 | 0.19 | 5.14 | 10 | 0.38 | 6.34 | 7 |
| Changes to labelling to promote healthier options at food outlets. | 0.22 | 5.04 | 10 | 0.19 | 5.17 | 11 | 0.23 | 6.18 | 7 | 0.25 | 4.19 | 11 |
| Changes to placement of healthier food options in neighbourhood grocery stores | 0.22 | 4.92 | 11 | 0.19 | 5.25 | 10 | 0.18 | 4.85 | 11 | 0.28 | 4.63 | 10 |
| Cooking classes to demonstrate affordable, healthy recipes for home cooking | 0.19 | 4.33 | 12 | 0.24 | 6.77 | 9 | 0.11 | 2.96 | 12 | 0.22 | 3.60 | 12 |
| Health educational resources to improve health knowledge | 0.15 | 3.49 | 13 | 0.27 | 7.54 | 6 | 0.04 | 1.18 | 14 | 0.14 | 2.28 | 13 |
| Changes to labelling of products in neighbourhood grocery stores | 0.09 | 2.17 | 14 | 0.02 | 0.46 | 14 | 0.22 | 5.93 | 9 | 0.07 | 1.15 | 14 |
| Total | 4.37 | 100.00 |  | 3.58 | 100.00 |  | 3.67 | 100.00 |  | 6.05 | 100.00 |  |

**** denotes p < .01, **p < .05, *p < .10*

### Table S4. Predicted and actual responses to holdout task question, by KRT

|  | **Total (n=1,453)**  % [95% CI] | **PPR Pekan Kepong (n=491)**  % [95% CI] | **PA Sri Negeri Sembilan (n=489)**  % [95% CI] | **PPR Seri Kota (n=473)**  % [95% CI] |
| --- | --- | --- | --- | --- |
| Predicted Percentage of Respondents Choosing Each Option in Holdout Task | | | | |
| Option A | 48%  [43%-51%] | 43%  [36%-51%] | 48%  [40%-55%] | 51%  [43%-58%] |
| Option B | 44%  [41%-49%] | 48%  [41%-55%] | 41%  [46%-48%] | 44%  [38%-52%] |
| Neither | 9%  [8%-9%] | 9%  [8%-10%] | 11%  [10%-13%] | 5%  [4%-6%] |
| Actual Percentage of Respondents Choosing Each Option in Holdout Task | | | | |
| Option A | 42% | 43% | 38% | 46% |
| Option B | 49% | 48% | 51% | 48% |
| Neither | 9% | 9% | 11% | 7% |
